# Complete mitochondrial genome of *Aedes flavopictus* (Yamada, 1921) (Diptera: Culicidae) collected in South Korea

**DOI:** 10.1080/23802359.2020.1863164

**Published:** 2021-01-27

**Authors:** Jiyeong Shin, Jongwoo Jung

**Affiliations:** a The Division of EcoCreative, Ewha Womans University, Seoul, Republic of Korea; b Department of Science Education, Ewha Womans University, Seoul, Republic of Korea

**Keywords:** *Aedes flavopictus*, invasive species, mitochondrial genome

## Abstract

In this study, we determined for the first time the mitochondrial genome sequence of an *Aedes flavopictus* specimen collected in South Korea. Its mitochondrial genome was 16,060 bp in length, consisting of 13 protein-coding, 22 tRNA, and 2 rRNA genes and a non-coding A + T rich region. The overall base composition in the heavy strand was 39.7, 8.6, 12.7, and 39% of A, G, C, and T, respectively, and the G + C content was 21.2%. Phylogenetic analysis revealed that *Aedes* spp. formed a monophyletic clade.

Mosquitoes belonging to the genus *Aedes* are responsible for transmitting fatal human diseases (Alphey et al. 2013). For example, *Aedes albopictus* and *Aedes aegypti* can carry and spread the Zika and yellow fever viruses (Lambrechts et al. 2010). *Aedes flavopictus* is widely distributed in East Asia, including Korea and Japan, belonging to the same genus as *A. albopictus* and *Aedes koreicus* (Tanaka 1979; Shin and Jung 2020). This particular mosquito had never been found in countries other than Korea and Japan until 2019, when it was identified in the Netherlands and a new invasion of Europe was confirmed (Doggett et al. 2019; Ibáñez-Justicia et al. 2019). No other vector is known to have the same characteristic as *A. albopictus* and other *Aedes* species; however, *A. flavopictus* has previously shown the potential to transmit dengue fever in laboratory conditions (Toma et al. 2002; Srisawat et al. 2016). In this study, the mitochondrial genome sequence of *A. flavopictus* (GenBank accession number: MT501510) was confirmed.

We collected an *A. flavopictus* specimen from Bonghwa-gun, South Korea (geographic location: 36°47′05.0′′N 128°56′32.6′′E) in June 2017. The specimen was preserved in 80% ethanol and stored at the Ewha Womans University Natural History Museum of Korea (accession number: EWNHM-DONATION-IN-2). The mtDNA was sequenced using Novaseq 6000 System (Illumina, San Diego, CA, USA); the MITObim method (Hahn et al. 2013) and MITOS (Bernt et al. 2013) were used for the assembly and annotation of the complete mitochondrial genome, respectively.

The mitogenome of *A. flavopictus* was 16,060 bp in length and consisted of 13 protein-coding, 22 tRNA, and 2 rRNA genes and a non-coding A + T-rich control region. For the 13 protein-coding genes, the most common shared start codon was ATG (in *COX2*, *ATP6*, *COX3*, *ND4L*, *ND4*, and *CYB*), followed by ATT (in *ND2*, *ND3*, *ND6*, and *ATP8*). The most common termination codon was TAA (in *ND2*, *ATP8*, *ATP6*, *COX3*, *ND3*, *ND5*, *ND4*, *ND4L*, *ND6*, *CYTB*, and *ND1*), followed by the incomplete termination codon T− (in *COX1* and *COX2*). The overall mitochondrial base composition of this genome was A: 39.7%, T: 39%, G: 8.6%, and C: 12.7%, with a G + C content of 21.2%.

To investigate whether *A. flavopictus* was genetically similar to other viral vector mosquitoes that carry the Zika and dengue fever viruses, its phylogenetic position within the family Culicidae was investigated using the complete mitochondrial genome sequence of nine species (Figure 1). The phylogenetic trees were constructed by MEGA X (Kumar et al. 2018) using the neighbor-joining algorithm with 1000 replicates. The results revealed that *Aedes* spp. formed a monophyletic group.

**Figure 1.**
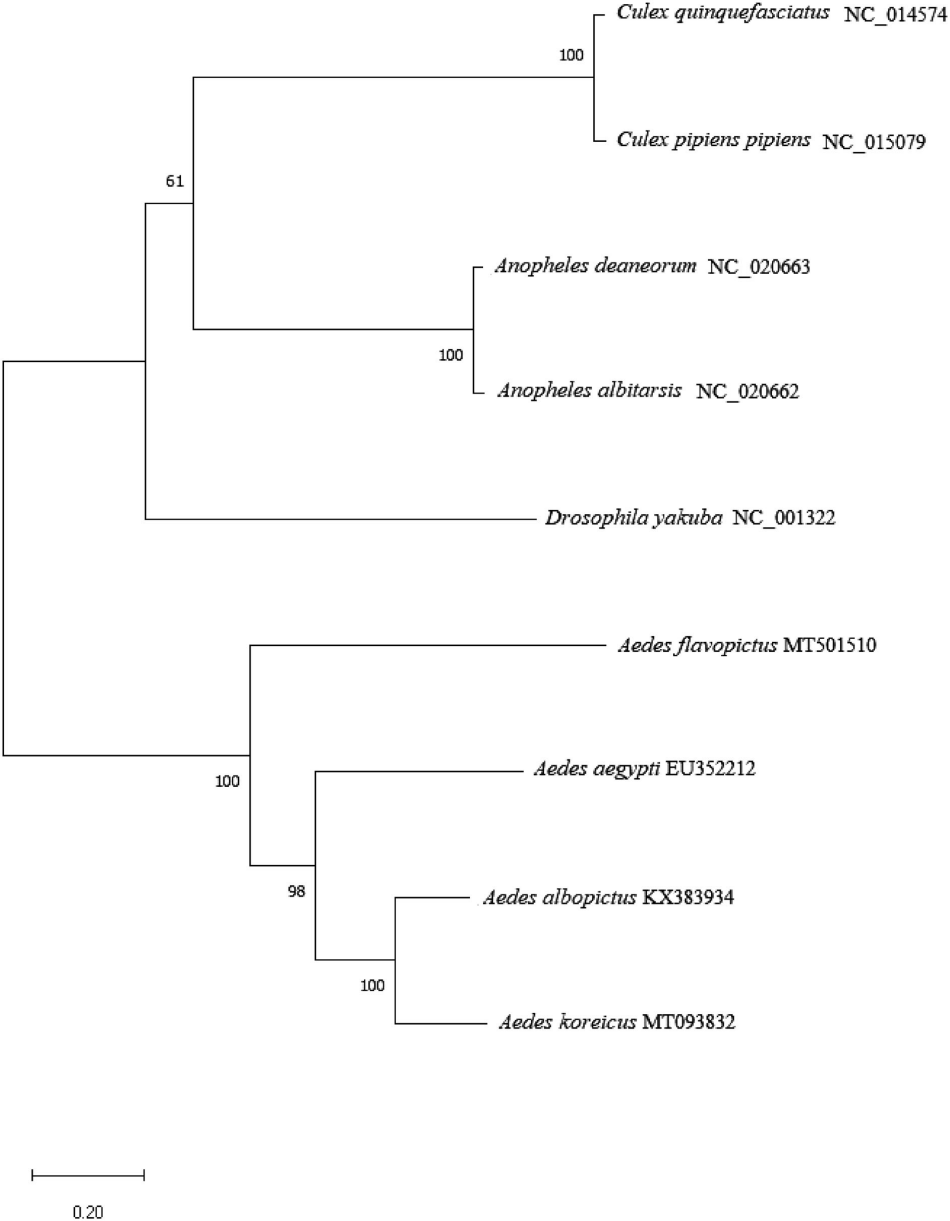
Phylogenetic tree of nine species within Culicidae constructed using the neighbor-joining algorithm based on complete mosquito mitochondrial genomes. Genbank Accession Numbers: *Aedes flavopictus* (MT501510), *Aedes koreicus* (MT093832), *Aedes albopictus* (KX383934), *Aedes Aegypti* (EU352212), *Anopheles albitarsis* (NC_020662), *Anopheles deaneorum* (NC_020663), *Culex pipiens pipiens* (NC_015079), *Culex quinquefasciatus* (NC_014574), and *Drosophila yakuba* (NC_001322).

These findings provide novel genetic insights into the members of the genus *Aedes* that could be relevant to better understand these vectors known to be responsible for spreading several deadly human diseases.

## Data Availability

The data that support the findings of this study are openly available in Mendeley Data at http://dx.doi.org/10.17632/b2mfmmdhnx.1
